# Early *E*. *casseliflavus* gut colonization and outcomes of allogeneic hematopoietic cell transplantation

**DOI:** 10.1371/journal.pone.0220850

**Published:** 2019-08-08

**Authors:** Armin Rashidi, Maryam Ebadi, Robin R. Shields-Cutler, Kathryn Kruziki, Dawn A. Manias, Aaron M. T. Barnes, Todd E. DeFor, Patricia Ferrieri, Jo-Anne H. Young, Dan Knights, Bruce R. Blazar, Daniel J. Weisdorf, Gary M. Dunny

**Affiliations:** 1 Division of Hematology, Oncology, and Transplantation, Department of Medicine, University of Minnesota, Minneapolis, MN, United States of America; 2 Division of Pediatric Hematology/Oncology, Department of Pediatrics, University of Minnesota, Minneapolis, MN, United States of America; 3 BioTechnology Institute, College of Biological Sciences, University of Minnesota, MN, United States of America; 4 Department of Biology, Macalester College, Saint Paul, MN, United States of America; 5 Department of Microbiology and Immunology, University of Minnesota, Minneapolis, MN, United States of America; 6 Department of Laboratory Medicine and Pathology, University of Minnesota, Minneapolis, MN, United States of America; 7 Biostatistics Core, Masonic Cancer Center, University of Minnesota, Minneapolis, MN, United States of America; 8 Division of Infectious Disease and International Medicine, Department of Medicine, University of Minnesota, Minneapolis, MN, United States of America; 9 Division of Blood and Marrow Transplantation, Department of Pediatrics, University of Minnesota, Minneapolis, MN, United States of America; University of Florida, UNITED STATES

## Abstract

Gut dysbiosis has been associated with worse allogeneic hematopoietic cell transplantation (allo-HCT) outcomes. We reported an association between intrinsically vancomycin-resistant enterococci (iVRE: *E*. *gallinarum* and *E*. *casseliflavus*) gut colonization and lower post-transplant mortality. In this study, using an expanded cohort, we evaluated whether our previously observed association is species-specific. We included allo-HCT recipients with ≥1 positive rectal swab or stool culture for iVRE between days -14 and +14 of transplant. To investigate whether iVRE modulate the gut microbiota, we performed agar diffusion assays. To investigate whether iVRE differ in their ability to activate the aryl hydrocarbon receptor, we analyzed iVRE genomes for enzymes in the shikimate and tryptophan pathways. Sixty six (23 *E*. *casseliflavus* and 43 *E*. *gallinarum*) of the 908 allograft recipients (2011–2017) met our inclusion criteria. Overall survival was significantly higher in patients with *E*. *casseliflavus* (91% vs. 62% at 3 years, *P* = 0.04). In multivariable analysis, *E*. *casseliflavus* gut colonization was significantly associated with reduced all-cause mortality (hazard ratio 0.20, 95% confidence interval 0.04–0.91, *P* = 0.04). While agar assays were largely unremarkable, genome mining predicted that *E*. *casseliflavus* encodes a larger number of enzymes in the tryptophan metabolism pathway. In conclusion, *E*. *casseliflavus* gut colonization is associated with reduced post-HCT morality. Further research is needed to understand the mechanisms for this association.

## Introduction

The importance of gut microbiota as a predictor of allogeneic hematopoietic cell transplantation (HCT) outcomes has become apparent in recent years [1]. We recently reported an unexpected association between pre-HCT gut colonization with intrinsically vancomycin-resistant enterococci (iVRE: *E*. *gallinarum* and *E*. *casseliflavus*) and improved overall survival (OS) due to decreased non-relapse mortality (NRM) [2]. iVRE are characterized by susceptibility to teicoplanin and constitutive low-level vancomycin resistance through the chromosomally encoded *vanC* genotype [3,4].

The objective of the present study was to determine whether our reported association between pre-HCT iVRE gut colonization and improved outcomes is species-specific (i.e., *E*. *gallinarum* vs. *E*. *casseliflavus*). In an expanded cohort, now also including patients who were iVRE colonized early after HCT, we demonstrate that this association with improved survival is specific to *E*. *casseliflavus*. Using in vitro agar diffusion assays and bioinformatic analysis of iVRE genomes, we explored two potential mechanisms for an *E*. *casseliflavus*-mediated effect: (*i*) a direct effect on the host and (*ii*) an indirect effect on the host via modulation of the gut microbiota.

## Materials and methods

### Patients

The University of Minnesota Institutional Review Board approved the study protocol. We retrospectively studied allo-HCT recipients at the University of Minnesota (2011–2017) who had ≥1 positive rectal swab or stool culture for iVRE between days -14 and +14. New admissions for HCT were screened for gut VRE colonization weekly until discharge. Spectra^TM^ VRE chromogenic agar medium (Thermo Fisher Scientific, Waltham, MA) was used for enterococcal culture. Species-level identification was done by Vitek 2 instrument and MALDI-TOF mass spectrometry (both from bioMerieux, Durham, NC) and antibiotic sensitivity were performed by broth microdilution from a subculture of the isolate using the colorimetric Vitek 2 automated system (bioMérieux, Durham, NC). Colonization level, classified as light, moderate, and heavy growth according to our clinical laboratory standards, was extracted from the patients’ electronic medical records. Antimicrobial prophylaxis consisted of levofloxacin, acyclovir, and an azole. Cefepime was our empiric antibiotic for neutropenic fever. OS was estimated using Kaplan-Meier curves. Relapse, grade II-IV acute graft-versus-host-disease (GVHD), chronic GVHD and NRM were estimated using cumulative incidence, treating NRM (for relapse), non-GVHD death (for GVHD), and relapse (for NRM) as competing risks. In the analysis of relapse and NRM, we excluded patients with non-malignant disorders. Fine and Gray regression was used to adjust for potential confounding [5]. All analyses were performed using SAS 9.4 (SAS Institute, Cary, NC).

## Agar diffusion assays

Intestinal domination may increase the risk of bacteremia and worsen transplant outcomes [6]. To investigate whether *E*. *casseliflavus* produces bacteriocins that create colonization resistance against prototype colonizers of the gut, we screened 16 *E*. *casseliflavus* clinical isolates for activity against 6 different *E*. *faecium* and *E*. *faecalis* isolates via agar diffusion assays. These *E*. *casseliflavus* isolates were provided by the clinical laboratory at our institution and retrieved from a repository of samples collected from inpatients (not necessarily HCT patients) over the last 10 years. In addition, a subset of the *E*. *casseliflavus* isolates were tested against two *E*. *coli* isolates, two *Streptococcus agalactiae* (group B) isolates, and a *Streptococcus gordonii* isolate.

A 300 μL aliquot of the indicated growth medium (M9YE [7] or Todd-Hewitt broth) was inoculated with enterococcal producer or indicator strain colonies and incubated for 18 hours at 37°C. The following day, indicator plates were prepared by inoculating 50 mL of warm medium + 1% w/v agar with 50 μL of an overnight culture of the indicator strain. Using a multichannel pipette, 1 μL of producer culture then was stabbed and released into the agar. Plates were then incubated for 18 hours at 37°C prior to imaging. Activity was determined based on the presence or absence of a clear zone of inhibition surrounding the producer strain inoculum.

### Bioinformatic analysis of shikimate/tryptophan pathways

Host-microbial crosstalk at the gut barrier is partially regulated by indole, indole-derived metabolites, and other shikimate/tryptophan pathway compounds, some of which are aryl hydrocarbon receptor (AhR) agonists with immunomodulatory and gut barrier protective functions [8,9]. To bioinformatically investigate whether *E*. *casseliflavus*, *E*. *gallinarum*, *E*. *faecium*, and *E*. *faecalis* may possess similar ARH agonist pathways, we analyzed the set of shikimate and tryptophan metabolism enzymes present in sequenced genomes from each species. A total of 15 *E*. *casseliflavus* (8 human isolates, of which 6 were disease-associated), 8 *E*. *gallinarum* (3 human disease-associated isolates), 11 *E*. *faecium* (10 human isolates, of which 8 were disease-associated), and 7 *E*. *faecalis* genomes (5 human isolates, of which 4 were disease-associated; for all species NCBI RefSeq latest assembly versions were used, accessed 3/19/2018; all classified as full genome representation) were annotated with PROKKA v1.12 [10] using default settings plus the following arguments: “—gcode 11—genus Enterococcus—species [casseliflavus]/[gallinarum]/[faeciuj]/[faecalis]—usegenus—kingdom Bacteria”. The annotations were then manually searched for enzymes present in shikimate and tryptophan metabolism as identified in the KEGG database [11]. The resulting presence/absence data was processed, ordered by unsupervised hierarchical clustering, and plotted using custom scripts in R (version 3.4; R Foundation for Statistical Computing, Vienna, Austria).

## Results

**Fig 1:**
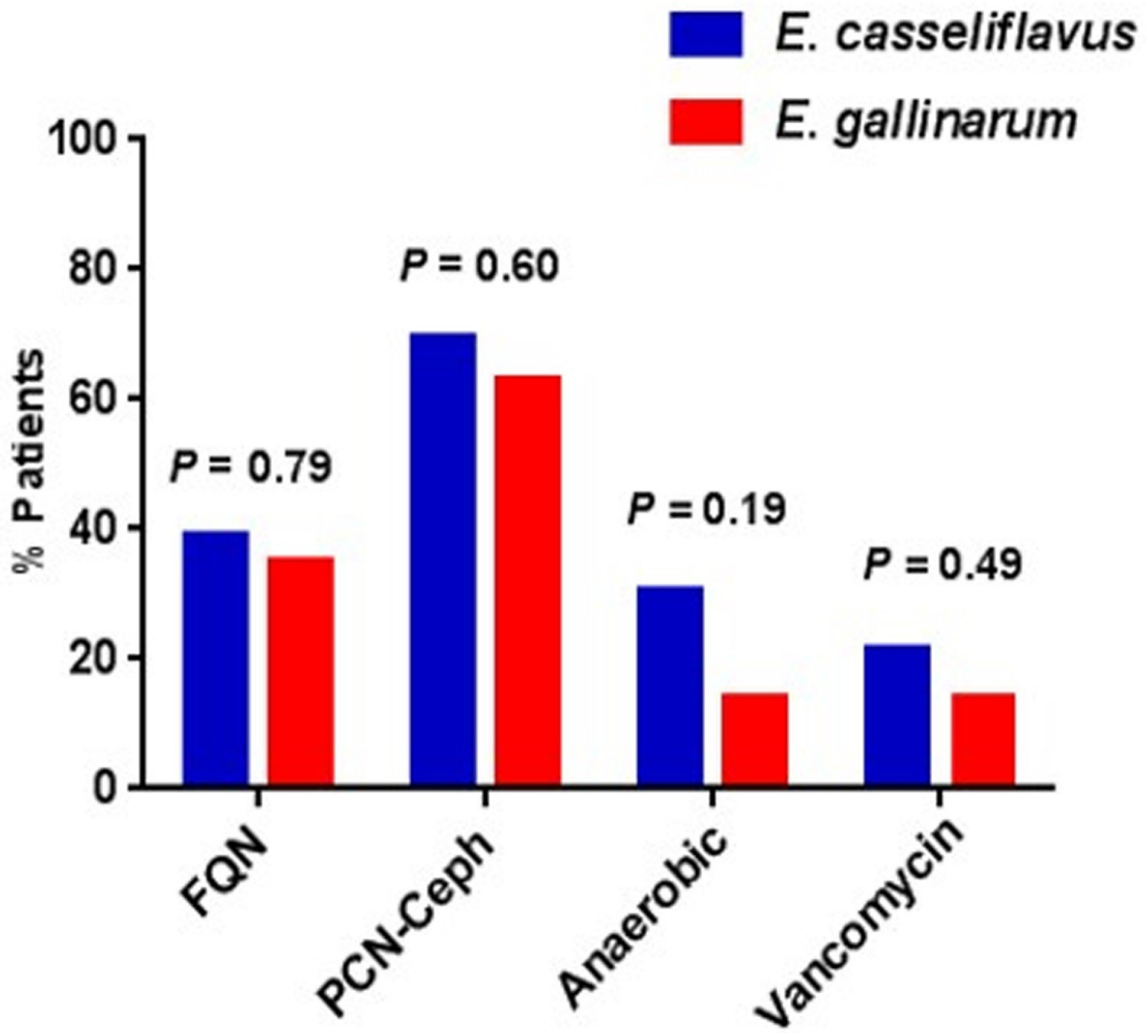
Antibacterial antibiotic exposure in patients with *E*. *casseliflavus* vs. *E*. *gallinarum* gut colonization. There were no significant differences between the groups. We considered antibiotic exposures starting 4 weeks before admission to the hospital until the colonization event. We classified antibiotics to four major groups that are commonly used before or shortly after HCT. Anaerobic: Antibiotics with potent anti-anaerobic activity (piperacillin-tazobactam, carbapenems, metronidazole, clindamycin); FQN: Fluoroquinolone; PCN/Ceph: Penicillins or Cephalosporins.

Sixty six (23 with *E*. *casseliflavus* and 43 with *E*. *gallinarum*) of the 842 allograft recipients between 2011–2017 met our inclusion criteria. As expected, since vancomycin resistance in iVRE is constitutive rather than acquired, the groups did not differ in their prior exposure to different classes of antibiotics (**Fig 1**). The abundance of iVRE in cultures were classified as light in 3 (13%), moderate in 7 (30%), heavy in 10 (43%), and not available in 3 (13%) patients with *E*. *casseliflavus* colonization. The abundance of iVRE in cultures were classified as light in 8 (19%), moderate in 9 (21%), and heavy in 26 (60%) in patients with *E*. *gallinarum* colonization. Nine (39%) patients colonized with *E*. *casseliflavus* and 34 (79%) of those colonized with *E*. *gallinarum* had at least one other sample colonized with the same species between days -14 and +14. These findings suggest that *E*. *gallinarum* colonization is more stable than *E*. *casseliflavus* colonization. Only 2 (9%) patients with *E*. *casseliflavus* and none with *E*. *gallinarum* colonization had VRE colonization between days -14 and +14. Five patients in each group (22% and 12%) had vancomycin-sensitive enterococcus (VSE) gut colonization between days -14 and +14.

**Fig 2:**
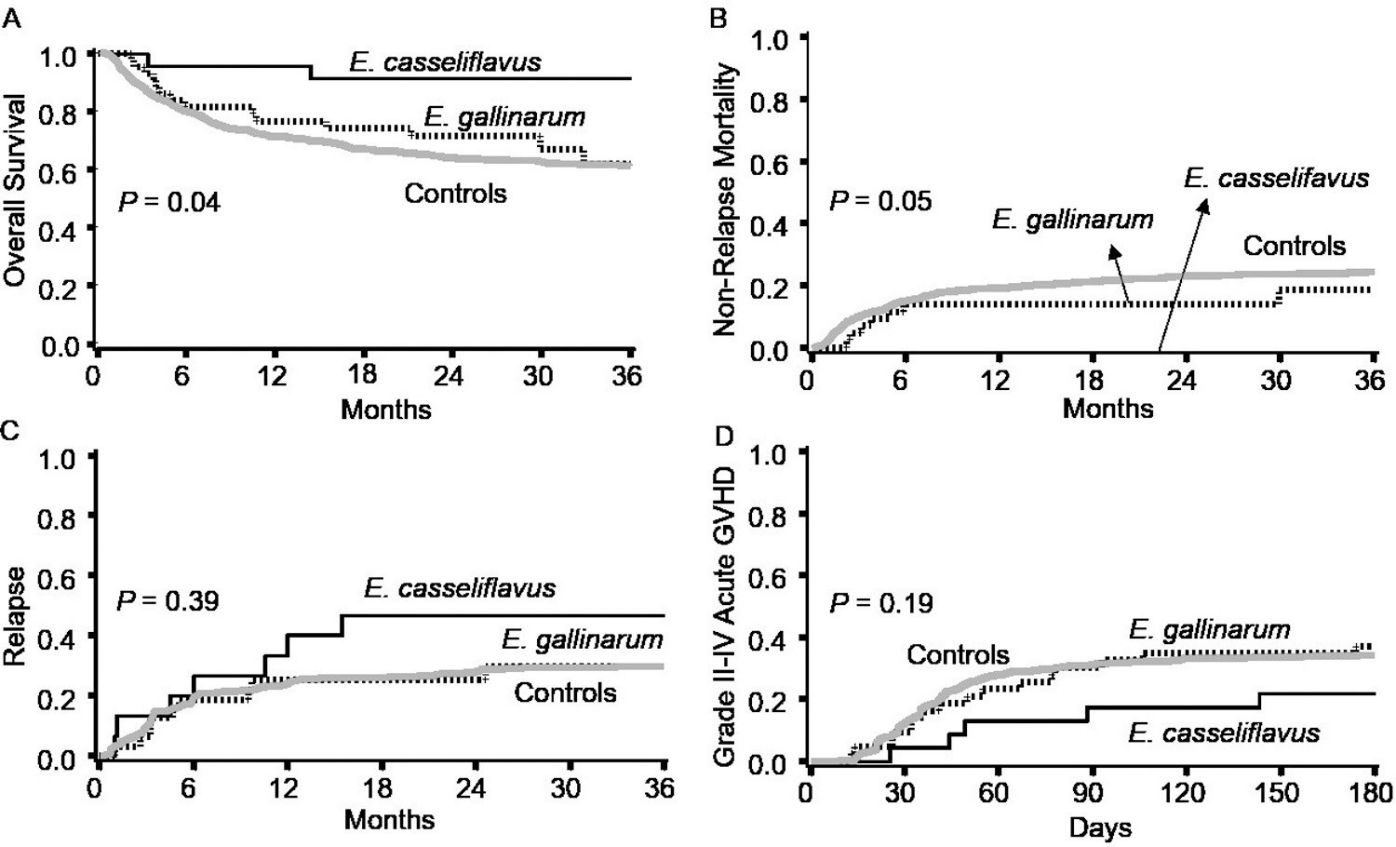
Comparison of outcomes between the groups with *E*. *casseliflavus* and *E*. *gallinarum* gut colonization. Patients with *E*. *casseliflavus* had a significantly higher overall survival (**A**), due to lower non-relapse mortality (**B**). The cumulative incidence of relapse (**C**) and grade II-IV acute GVHD (**D**) were not significantly different between the groups. Curves for a control group of 776 patients who did not develop *E*. *casseliflavus* or *E*. *gallinarum* gut colonization are also shown, with outcomes not different from the *E*. *gallinarum* group. *P* values compare patients with *E*. *casseliflavus* vs. *E*. *gallinarum*. aGVHD: Acute graft-versus-host disease.

There were no significant differences between the groups in patient-, disease-, or transplant-related characteristics, except a higher frequency of sirolimus-based GVHD prophylaxis in the *E*. *casseliflavus* group (35% vs. 12%, *P* = 0.05; **Table 1**). **Fig 2**compares transplant outcomes between the groups with *E*. *casseliflavus* gut colonization, *E*. *gallinarum* gut colonization, and the 776 control patients who did not have colonization with either species. The median follow up for the first two groups was 30 (interquartile range 22–41) months. No deaths occurred before day +14. Comparing patients with *E*. *casseliflavus* vs. *E*. *gallinarum* colonization, OS was significantly higher in patients with *E*. *casseliflavus* (91% vs. 62% at 3 years, *P* = 0.04), due to lower NRM (0 vs. 18% at 3 years, *P* = 0.05). Only two patients with *E*. *casseliflavus* died within 3 years post-HCT, both due to relapse. In contrast, 14 patients with *E*. *gallinarum* died within 3 years post-HCT: 7 (50%) due to relapse and 7 (50%) from NRM (4 GVHD, 1 infection, 1 graft failure, and 1 veno-occlusive disease). Relapse rates were not significantly different between the groups (47% vs. 30%, *P* = 0.39). 3-year rates of OS, NRM, relapse, and grade II-IV acute GVHD among controls were 61% (95% confidence interval 57%-64%), 24 (21–27)%, 30 (26–34)%, and 34 (31–37)%, respectively. These rates were similar to the *E*. *gallinarum* group.

**Table 1:** Baseline patient-, disease-, and transplant-related characteristics.

			*E*. *casseliflavus*	*E*. *gallinarum*	*P*	*Controls*
**N**			23	43	-	776
**Gender, n (%)**					0.38	
	Male		13 (57)	29 (67)\		469 (60)
	Female		10 (43)	14 (33)		307 (40)
**Age, median (range)**			10 (1–66) years	28 (1–72) years	0.32	38 (<1–74) years
**Donor, n (%)**					0.76	
	HLA-matched sibling		5 (22)	12 (28)		277 (35)
	Umbilical cord blood		15 (65)	24 (56)		316 (41)
	Others		3 (13)	7 (16)		172 (22)
		HLA-haploidentical	3	4		19 153
		Unrelated donor	0	3		
**Conditioning intensity, n (%)**					1.00	
	Myeloablative		12 (52)	23 (53)		339 (44)
		TBI-based	6	15		225
		Non-TBI-based	6	8		114
	Reduced-intensity		11 (48)	20 (46)		437 (56)
**GVHD prophylaxis, n (%)**					0.05	
	Calcineurin inhibitor-based		15 (65)	38 (88)		686 (88)
	Sirolimus-based		8 (35)	5 (12)		90 (12)
**Underlying disease, n (%)**					0.73	
	Acute leukemia		9 (39)	20 (47)		344 (44)
		AML	6	15		227
		ALL	3	5		117
	Other malignant		6 (26)	12 (27)		220 (28)
		MDS/MP	1	4		107
		Lymphoma/CLL	3	7		94
		Myeloma	2	1		19
	Non-malignant		8 (35)	11 (26)		212 (27)
**Disease status at HCT, n (%)**					0.59	
	CR		12 (52)	28 (65)		407 (52)
	Not CR		3 (13)	4 (9)		157 (20)
	Non-malignant		8 (35)	11 (26)		212 (27)
**Disease risk index, n (%)**					0.62	NA
	Low		2 (9)	4 (9)		
	Intermediate		6 (26)	18 (42)		
	High/Very high		7 (30)	10 (23)		
	Non-malignant		8 (35)	11 (26)		
**HCT comorbidity index, n (%)**					0.87	
	0		11 (48)	23 (53)		344 (45)
	1–2		9 (39)	14 (33)		246 (32)
	>2		3 (13)	6 (14)		186 (24)
**Follow up, median (interquartile range)**			26 (22–40)	29 (22–43)	0.15	42 (24–59)
**iVRE-to-HCT interval, median (range)**			-1 (-9 to 13) days	-3 (-13 to 14) days	0.14	-

The control group consists of all other stem cell allograft recipients at the University of Minnesota (2011–2017). ALL: Acute lymphoblastic leukemia; AML: Acute myeloid leukemia; CLL: Chronic lymphocytic leukemia; CR: Complete remission; GVHD: Graft-versus-host disease; HCT: Hematopoietic cell transplantation; HLA: Human leukocyte antigen; iVRE: Intrinsically vancomycin-resistant enterococci; MDS: Myelodysplastic syndromes; MPN: Myeloproliferative neoplasms; NA: Not available; TBI: Total body irradiation

In multivariable analysis, *E*. *casseliflavus* gut colonization was significantly associated with reduced all-cause mortality (hazard ratio 0.20, 95% confidence interval 0.04–0.91, *P* = 0.04). There were no significant differences between the groups in 180-day grade II-IV acute GVHD (22% in *E*. *casseliflavus* vs. 37% in *E*. *gallinarum*; *P* = 0.19), 1-year chronic GVHD (9% vs. 12%, *P* = 0.56), 100-day bacteremia (22% vs. 28%, *P* = 0.59), or 100-day *Clostridium difficile* infection (17% vs. 21%, *P* = 0.79). The groups did not differ in time to platelet recovery (median 42 vs. 38 days, *P* = 0.46) but time to neutrophil recovery was longer in the *E*. *casseliflavus* group (median 16 vs. 12 days, *P* = 0.02).

**Table 2**shows the results of agar diffusion assays measuring susceptibility of well-characterized other enterococcal species that might be competitors in the gut to potential bacteriocins or other compounds secreted by *E*. *casseliflavus* or *E*. *gallinarum*. Other than one *E*. *casseliflavus* isolate (#10 in **Table 2**), which had potent activity against both *E*. *faecium* and *E*. *faecalis*, no other *E*. *casseliflavus* isolates showed consistent zones of inhibition. We found no consistent differences between the two growth media tested in these assays. To examine the possibility that *E*. *casseliflavus* might produce bacteriocins or other compounds that target potentially-competitive non-enterococcal bacteria strains in the gut, we also tested five additional clinical isolates via the agar diffusion assay—two *E*. *coli* isolates, two *Streptococcus agalactiae* isolates, and a *Streptococcus gordonii* isolate–against a subset of six of the *E*. *casseliflavus* strains noted in **Table 2**. None of the non-enterococcal isolates were affected by the *E*. *casseliflavus* strains tested. These results suggest that a competitive advantage over other bacteria did not contribute to *E*. *casseliflavus* dominance in the intestines of patients with improved survival.

**Table 2:** Sensitivity of *E*. *faecium* and *E*. *faecalis* to potential *E*. *casseliflavus* bacteriocins as measured by agar diffusion assays.

	*E*. *faecium* 8E9	*E*. *faecium* 9B	*E*. *faecium* 7A	*E*. *faecium* 6E6	*E*. *faecalis* OG1RF	*E*. *faecalis* V583
Isolate 1						
Isolate 2						
Isolate 3						
Isolate 4						
Isolate 5						
Isolate 6						
Isolate 7						
Isolate 8						
Isolate 9						
Isolate 10						
Isolate 11						
Isolate 12						
Isolate 13						
Isolate 14						
Isolate 15						
Isolate 16						
*E*. *faecium* 8E9						
*E*. *faecium* 9B						
*E*. *faecium* 7A						
*E*. *faecium* 6E6						
*E*. *faecalis* OG1RF						
*E*. *faecalis* V583						

Sixteen clinical isolates of *E*. *casseliflavus* (first column) were tested as potential bacteriocin producers against indicator strains of *E*. *faecium* and *E*. *faecalis* (first row). Included in the first column are the same *E*. *faecium* and *E*. *faecalis* strains, now used as producers. Shading indicates bacteriocins sensitivity.

**Fig 3:**
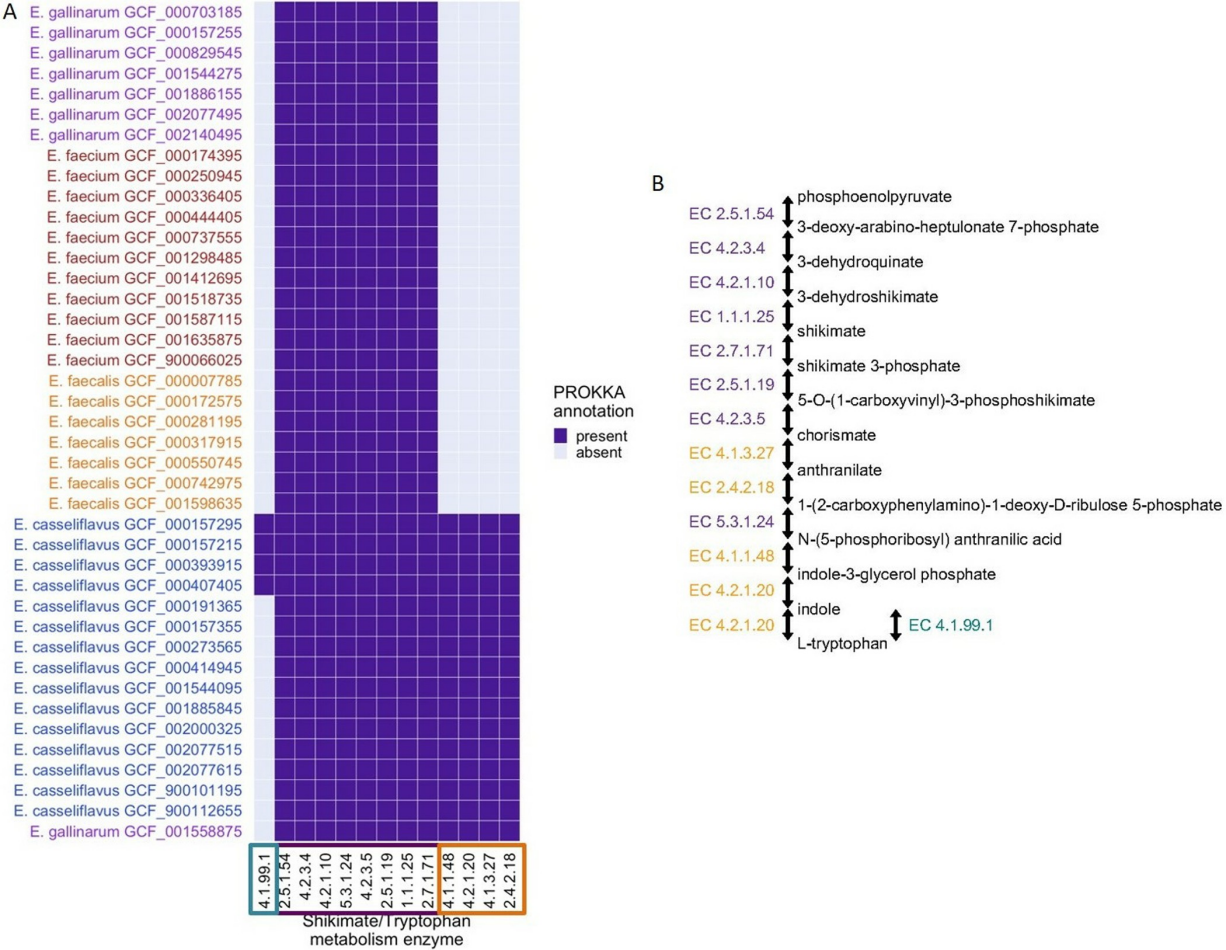
Bioinformatic analysis of 41 publicly available *Enterococcus* genomes for 14 enzymes in shikimate and tryptophan metabolism pathways. Predicted presence/absence matrix for each enzyme (shikimate and tryptophan metabolism pathways) found in at least one of the 41 sequenced *Enterococcus* genomes, with pathways and isolate genomes arranged by unsupervised hierarchical clustering (**A**). Simplified metabolic pathway demonstrating the two routes to indole production (via chorismate and via tryptophan) (**B**). 4.2.1.10: 3-dehydroquinate dehydratase; 1.1.1.25: shikimate dehydrogenase; 2.7.1.71: shikimate kinase; 2.5.1.19: 3-phosphoshikimate 1-carboxyvinyltransferase; 4.2.3.5: chorismate synthase; 4.1.327: anthranilate synthase; 2.4.2.18: anthranilate phosphoribosyltransferase; 5.3.1.24: phosphoribosylanthranilate isomerase; 4.1.1.48: indole-3-glycerol-phosphate synthase; 4.2.1.20: tryptophan synthase; 1.2.1.3: aldehyde dehydrogenase (NAD+); 4.1.99.1: tryptophanase; 4.2.3.4: 3-dehydroquinate synthase; 2.5.1.54: 3-deoxy-7-phosphoheptulonate synthase.

Our bioinformatic analysis of 41 publicly available *Enterococcus* genomes for enzymes in shikimate and tryptophan metabolism pathways predicted that *E*. *casseliflavus* encodes a larger number of enzymes in the tryptophan metabolism pathway (**Fig 3**). This analysis predicted that four enzymes in the chorismate pathway to indole are present only in *E*. *casseliflavus*. In addition, tryptophanase, which converts tryptophan to indole, was predicted to be present in four *E*. *casseliflavus* genomes but none of the other *E*. *casseliflavus* or non-*casseliflavus* genomes.

Because of the known role of indole in AhR agonism [12,13], potential involvement of the four casseliflavus-specific enzymes in indole production, and the availability of a sensitive and specific assay for indole production [14], we tested 16 diverse *E*. *casseliflavus* strains, and selected isolates of *E*. *gallinarum*, *E*. *faecium*, and *E*. *faecalis* for indole production in vitro (**Methods in S1 File**). None of the enterococcal isolates produced detectable indole in these assays, while a control *Escherichia coli* strain produced abundant indole under the same conditions (**Fig A in S1 File**).

## Discussion

The presence of *Blautia*, butyrate-producing *Clostridia spp* [15], and *Eubacterium limosum* [16] in the stool has been associated with reduced mortality after allo-HCT. Our results indicate a similar association for *E*. *casseliflavus*. Notably, although both *E*. *casseliflavus* and *E*. *gallinarum* are motile enterococci with intrinsic vancomycin resistance and have 74% average nucleotide identity [17], our results from this and our previous study show that the association between iVRE and reduced mortality after allo-HCT is specific to *E*. *casseliflavus*.

We explored two possibilities to explain the association between *E*. *casseliflavus* and reduced post-allo-HCT mortality. First, we hypothesized that *E*. *casseliflavus* may have a competitive advantage over pathobionts known to cause clinical infections in transplant recipients. Intestinal domination by pathogenic bacteria such as those with antibiotic resistance genes (*e*.*g*., VRE), coupled with a compromised gut barrier due to transplant conditioning and intestinal GVHD, may increase the risk of bloodstream infection [6,18]. Bacteriocin production augments enterococcal niche competition in the mammalian gut [19]. However, our results did not identify *E*. *casseliflavus* as a powerful producer of bacteriocins against *E*. *faecium* and *E*. *faecalis* (including 5 VRE and 1 vancomycin-sensitive strains). Similarly, *E*. *casseliflavus* did not have a competitive advantage against several other gut pathobionts in our agar diffusion assays.

Alternatively, *E*. *casseliflavus* may produce metabolites that directly benefit the host. One such metabolite is the commensal microbe-derived compound butyrate, which has both local and systemic immunomodulatory effects [20,21], and improves gut barrier integrity [15,22,23]. Another example is indole, a molecule produced from the catabolism of dietary tryptophan by commensal bacteria of the gut [24]. We chose to measure indole because indole and its derivatives have a wide range of immune- and non-immune-mediated effects on the host. Indoleamine 2,3-dioxygenase (an indole derivative) has tolerogenic activity and reduces acute GVHD lethality by causing T-cell anergy [25,26]. In a recent study, indole-3-carboxaldehyde limited gut barrier damage, reduced bacterial translocation, and decreased inflammatory cytokine production, reducing GVHD pathology and its mortality [12]. GVHD and complications of gut barrier injury such as bloodstream infection are the most common causes of NRM in HCT recipients. Our in vitro comparison of *E*. *casseliflavus* with other enterococci for indole production was unremarkable. However, indole is only one of the many AhR agonists produced via shikimate and tryptophan pathways. Investigating the other metabolites involved in the shikimate/tryptophan pathway may be informative.

Using a genome mining approach, we predicted the potential presence of more tryptophan pathway metabolites and AhR ligands in *E*. *casseliflavus* compared to *E*. *gallinarum*. AhR signals augment the gut barrier and modulate the immune system, potentially reducing pathogenic injury and inducing protective immune responses [27–30]. Although NRM was lower in *E*. *casseliflavus* patients, no specific cause of NRM was identified as the primary driver of this difference. We chose AhR ligands for our bioinformatic analysis because of their broad range of effects on the host and hence potential to explain the overall reduced NRM rates in *E*. *casseliflavus* patients. Our genomic prediction needs biochemical validation in future work.

Although iVRE can be isolated from stool in ~5% of hospitalized patients, clinical infections are rare [31–35]. Gut colonization with *E*. *casseliflavus* may not be in the causal pathway to improved transplant outcomes. Instead, it may be a surrogate for a healthier microbiome permitting colonization by a less pathogenic organism. Future metagenomics studies may identify microbiota signatures of *E*. *casseliflavus*-colonized samples and predict metabolic pathways in which these samples are enriched.

## Supporting information

S1 File Methods and Fig A. Lack of indole in culture supernatants of enterococcal strains.
(PDF)
