# Supplementary material for: Early E. casseliflavus gut colonization and outcomes of allogeneic hematopoietic cell transplantation
Source: PLoS One. 2019 Aug 8;14(8):e0220850. doi: 10.1371/journal.pone.0220850 (PMC6687141; doi:10.1371/journal.pone.0220850)
Supplement: S1 File — Lack of indole in culture supernatants of enterococcal strains. (PDF) [file pone.0220850.s001.pdf]

Methods: We examined indole production by 20 enterococcal strains as well as by *Escherichia coli* strain ATCC 8739 in vitro using the hydroxylamine-based assay described by Darkoh et al (1). The enterococci included 6 *E. casseliflavus* isolates, 1 *E. gallinarum* isolate, and two VanA$^+$ *E. faecium* isolates from the University of Minnesota Clinical Microbiology Laboratory, as well as 10 *E. casseliflavus* isolates from the Minneapolis Veterans Affairs hospital (2). In addition, the well-characterized *E. faecalis* OG1RF strain (3) was also tested.

Bacterial cultures (5 ml) inoculated from single colonies on agar plates were grown for approximately 25 h at 37°C in Brain-Heart Infusion medium, the cells were pelleted by centrifugation and 100 μL of each supernatant was mixed sequentially with 25 μL 5.3M NaOH followed by 50 μL NH_2OH-HCl. The mixtures were incubated for 15 min., followed by addition of 125 μL 2.7M H_2SO_4. After 30 min. the A_{530} was read on a BIO-TEK Synergy H1 Hybrid plate reader (for selected reactions, the plate reader values were confirmed spectrophotometrically). For two selected *E. casseliflavus* strains (gold bars below), we grew replicate cultures where the medium was supplemented with 0.5% tryptophan; the extra tryptophan did not increase indole production. To confirm the results we also spotted the bacteria on paper soaked in Kovac's reagent (REMEL), which replicates the indole assay generally used in clinical labs; these assays agreed with the liquid assays in all cases. Fig. S1 depicts the assay results, where the values represent the mean of 3 replicates; error bars are not shown, but were extremely small.

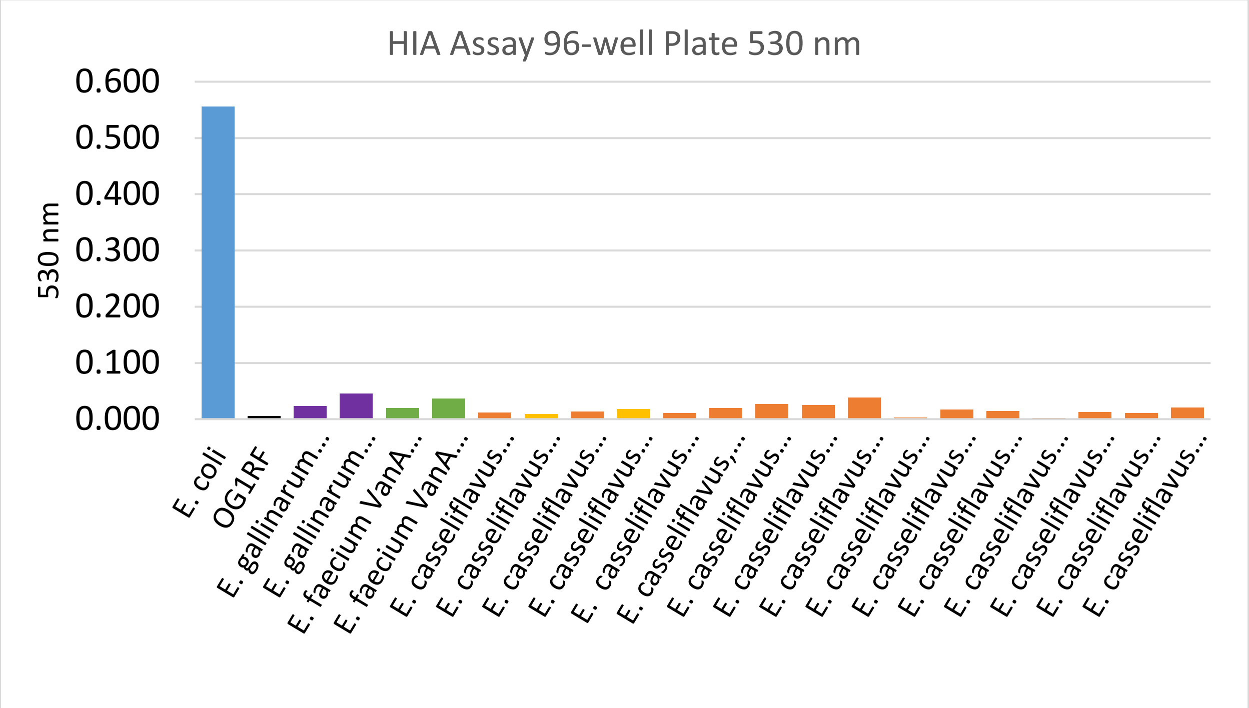

Fig A

References

1. Darkoh C, Chappell C, Gonzales C, Okhuysen P. 2015. A rapid and specific method for the detection of indole in complex biological samples. Appl Environ Microbiol 81:8093–7.
2. Leuck A-M, Johnson JR, Dunny GM. 2014. A widely used in vitro biofilm assay has questionable clinical significance for enterococcal endocarditis. PLoS One 9:e107282.
3. Bourgogne A, Garsin DA, Qin X, Singh K V, Sillanpaa J, Yerrapragada S, Ding Y, Dugan-Rocha S, Buhay C, Shen H, Chen G, Williams G, Muzny D, Maadani A, Fox KA, Gioia J, Chen L, Shang Y, Arias CA, Nallapareddy SR, Zhao M, Prakash VP, Chowdhury S, Jiang H, Gibbs RA, Murray BE, Highlander SK, Weinstock GM. 2008. Large scale variation in Enterococcus faecalis illustrated by the genome analysis of strain OG1RF. Genome Biol 9:R110.
